# Development and laboratory validation of a plant-derived repellent blend, effective against *Aedes aegypti* [Diptera: Culicidae], *Anopheles gambiae* [Diptera: Culicidae] and *Culex quinquefasciatus* [Diptera: Culicidae]

**DOI:** 10.1371/journal.pone.0299144

**Published:** 2024-03-21

**Authors:** Martyn J. Wood, James C. Bull, Kanagasooriyam Kanagachandran, Tariq M. Butt

**Affiliations:** 1 Department of Biosciences, Faculty of Science and Engineering, Swansea University, Swansea, United Kingdom; 2 Rentokil Initial 1927 plc, Crawley, West Sussex, United Kingdom; PLOS ONE, UNITED KINGDOM

## Abstract

Mosquitoes of the genera *Aedes*, *Anopheles* and *Culex* vector a wide range of pathogens seriously affecting humans and livestock on a global scale. Over-reliance on insecticides and repellents has driven research into alternative, naturally-derived compounds to fulfil the same objectives. Steam distilled extracts of four plants with strong, yet attractive, volatile profiles were initially assessed for repellency in a dual-port olfactometer using *Aedes aegypti* as the model species. *Picea sitchensis* was found to be the most repellent, proving comparable to leading products when applied at 100% (p = 1.000). Key components of conifer-derived volatile profiles were then screened via electroantennography before those components eliciting an electrophysiological response were assayed individually in the olfactometer; according to WHO protocol. The most promising 5 were selected for reductive analyses to produce an optimised semiochemical blend. This combination, and a further two variations of the blend, were then progressed to a multi-species analysis using the BG-test whereby bite-attempt frequency on hands was assessed under different repellent treatments; assays were compared between *Aedes aegypti*, *Anopheles gambiae* and *Culex quinquefasciatus*. Efficacy was found against all three species, although it was found that *Ae. aegypti* was the most susceptible to the repellent, with *An. gambiae* being the least. Here, a novel, naturally-derived blend is presented with weak spatial repellency, as confirmed in laboratory assays. Further work will be required to assess the full extent of the potential of the products, both in terms of field application and species screening; however, the success of the products developed demonstrate that plant metabolites have great capacity for use in the repellent sector; both to improve upon known compounds and to reduce the usage of toxic products currently on the market.

## 1. Introduction

The mosquitoes *Aedes aegypti* and *Aedes albopictus* are vectors of a wide range of viral diseases (e.g. Zika, Chikungunya, Dengue, Yellow Fever) which threaten the health of billions of people worldwide [1–5]. Dengue is recognised as one of the fastest-emerging infectious diseases globally, with 100–400 million new infections a year [6]. The impact of COVID-19 on dengue has been to produce an inverse relationship of infection [7], however, with a gradual return to ‘normal’ human behaviours, spread is likely to increase again, similar patterns may emerge for other arboviral diseases also. Compounding this, the geographic range of *Aedes* mosquitoes has expanded significantly due to climate change, expansion of trade and increased international travel [8–11]. The success of *Ae. aegypti* and *Ae. albopictus* as vectors can also be attributed to their ability to breed in disparate habitats including discarded bottles, cans, and other man-made containers, and the production of desiccation-resistant eggs [12]. These issues are further compounded by the development of insecticidal resistance in *Aedes* populations [13–16] and emergent evolutionary insensitivity to DEET [17]. Prevention of *Aedes*-transmitted diseases is dependent not only on deployment of effective vector control programmes including traditional insecticides, biologicals and sterile insect techniques [18, 19], but also use of personal preventative measures including bed nets and chemical repellents [20].

Female mosquitoes use olfaction while host-seeking for suitable vertebrate hosts from which to take a blood meal. Insect repellents disrupt olfactory processes, ultimately reducing host-seeking behaviour, landing, and subsequently bites. Commercial insect repellents are broadly divided into synthetic and natural repellents. The former include DEET (N, N-diethyl-3-methylbenzamide), IR3535 (3-(N-n-butyl-N-acetyl)-amino-propionic acid ethyl ester), and picardin (2-(2-hydroxyethyl)-1-methylpropylstyrene 1-piperidine carboxylate). Products containing natural plant-based repellents include p-menthane-3,8-diol (PMD) and citronella derived from lemon-scented eucalyptus (*Corymbia citriodora* [Myrtales: Myrtaceae]) and citronella grass (*Cymbopogon nardus* [Poales: Poaceae]), respectively [21]. DEET is one of the most widely used and efficacious of mosquito repellents, but there are conflicting reports on its potential risks for human health, especially in children [22]. There are also reports of DEET insensitivity developed in *Ae. aegypti* following prior exposure to the repellent [23].

Plants have historically been used as traditional repellents of hematophagous insects, with laboratory studies demonstrating that such plants often have varying degrees of success in repelling mosquitoes [1, 24, 25]. Most often, repellency is due to specific compounds, especially terpenoids [26–29], produced and contained within the plant matter. Limonene, 1,8-cineole, geraniol, citronella and eugenol are often listed among the main natural repellents found in plants [21, 27], each of which has received a significant degree of commercial interest and development.

To date, most studies have focussed on angiosperms, not gymnosperms, as a source of repellent compounds; no doubt due to their highly diverse volatile bouquets [30] and utility as attractants due to mosquito plant-sugar feeding behaviours [31, 32]. Of the limited studies on gymnosperm-derived extracts, most attention is focused on their larvicidal properties [29] with very few demonstrating repellency [33]. The current study reports on individual compounds, and blends thereof, derived from Sitka spruce, *Picea sitchensis* [Pinales: Pineaceae] in the scope of the development of a highly effective blend that causes significant repellency to a range of mosquito species under laboratory conditions. The blend has a pleasant perfume whilst retaining mosquito repellent properties, therefore, it has the potential to be used to control host-seeking mosquitoes by being developed into a product suitable for deployment in puffer sprays or applied to surfaces (e.g. clothing, bed nets, insect screens) or integrated into polymers for controlled release.

## 2. Materials and methods

### 2.1 Mosquitoes

Initial olfactometry and cage assays were conducted using *Aedes aegypti* (Strain: *AeAe*) raised from eggs procured from London School of Hygiene and Tropical Medicine (LSHTM, UK). Promising blends were then assayed in secondary tests against *Ae. aegypti* (strain: Rockefeller), *Culex quinquefasciatus* (strain: Biogenius), and *Anopheles gambiae* (strain: KISUMU), each of which having been reared onsite at Biogents (BG).

Prior to the start of any study, unfed adult females between three and seven days post emergence, starved for 24 hours prior to experimentation, were selected for their attraction to a volunteer’s arm held alongside a Bugdorm-4S4545 (Watkins and Doncaster, Leominster, UK) mesh insect rearing cage. Only those females responding quickly (< 30 seconds) were selected for use in assays.

### 2.2 Extraction of essential oils

Potted saplings of Sitka spruce (*Picea sitchensis*), (Maelor Forest Nurseries, UK), were maintained outdoors until required. The essential oils of *P. sitchensis* were compared in initial assays with extracts of three herbs, lavender (*Lavendula angustifolia* [Lamiales: Lamiaceae]), murihani (*Ocimum suave*, [Lamiales: Lamiaceae]), and rosemary (*Rosmarinus officinalis* [Lamiales: Lamiaceae]) all of which are known to contain mosquito repellent compounds [34–36]. The potted herbs were maintained in a glasshouse at 27 ± 4°C.

Essential oil extracts of the four plant species were prepared using a combination of steam distillation and solvent extraction [37]. Briefly, 400 g of leaf material was homogenized using a blender (Waring Commercial, Stamford, CT, USA) and the homogenate steam distilled using 500 mL distilled water. The condensed liquid was separated into 100 mL samples to which 50 mL of diethyl ether (99.99%, Fisher Scientific) was added. The mixture was gently agitated for 2 mins before the water was removed using a separation funnel. This process was repeated 6 times for each 100 mL condensate collected. The diethyl ether was removed by heating the sample in a water bath at 38°C until a layer of extracted oil remained. The process was repeated 3 times for each plant species. The total oil extracted for spruce, murihani, lavender and rosemary was 2.87 g, 3.04 g, 2.19 g and 6.34 g, respectively. Of these, 1 g was suspended in 1 mL hexane (>99.99% Sigma Aldrich) and represented 100% w/v of stock solution for initial olfactometry and repellency studies.

### 2.3 Test chemicals

The constituents of Sitka spruce essential oils have already been described [37, 38]. Thirteen compounds were selected for investigation as repellents of *Ae. aegypti*. These included: acetic acid, (+)-borneol, (-)-bornyl acetate, camphor, (-)-transcaryophelene, 1,8 cineole, 2-cyclohexen-1-ol, eugenol, eugenyl acetate, isoeugenol, α-pinene, β-pinene, α-terpineol. Controls included DEET and citronella. All chemicals were purchased from Sigma-Aldrich unless indicated otherwise and generally had >98% purity. For the BG cage assays, the controls included ethanol and commercial formulations of DEET (Anti Brumm® Forte 30% w/w, Hermes Arzneimittel GmbH, Deutschland), Picaridin (Autan® Protection Plus 20% w/w, SC Johnson GmbH, Deutschland), PMD (Anti Brumm® Naturel, 20% w/w, Hermes Arzneimittel GmbH, Deutschland), and IR3535 (Jaico Muggenmelk Natural, 19.6% w/w, Omega pharma Nederland BV, The Netherlands).

### 2.4 Essential oil olfactometry

Olfactometry was conducted using a modified Perspex dual port olfactometer [39] and only differed from the WHO 2013 [40] host attraction-inhibition assay by reducing the extract volume to 50 μL and increasing the mosquito numbers to 20.

Initial olfactometry assays were conducted using a 50 μL aliquot of each of the plant extracts, absorbed into filter paper measuring 20 x 20 mm and folded every 5 mm to aid evaporation as outlined earlier (see section 2.2). Concentrations of each extracted essential oil, along with DEET (Jungle Formula 50% DEET, Perrigo, UK) and citronella (100% Holland & Barrett, UK), were tested at 100% (1g mL^-1^) dose or a slightly diluted 75% (0.75g mL^-1^) dose. Nylon socks, worn for 24 hours prior to experimentation are proven mosquito lures and were used as a host odour source [41, 42]. A single sock was placed in one test-port of the olfactometer in addition to a filter paper containing either a treatment compound such as an essential oil, or hexane only which was used as the negative control. Treatments and controls were introduced to the test ports of the olfactometer after the 20 mosquitoes in the holding chamber had acclimatised for 15 mins. The air speed was 0.2 m s^-1^ in the test ports and 0.4 m s^-1^ in the release port. There were ten replicates per treatment. Results were determined as a percentage of the total number of mosquitoes attracted to treatment ports in each experiment. Repellency was indicated through the differential percentage attraction of mosquitoes to tests ports containing either host odours or a combination of the host odours and prospective repellent compounds, and those that sought refuge within the volatile free port of the olfactometer.

### 2.5 Electroantennography

To establish which of the selected spruces volatiles triggered excitatory responses in *Ae. aegypti* electroantennography (EAG) was performed Abdullah *et al*. 2014 [43]. The head of a 3-day old adult female *Ae. aegypti* (AeAe), that had not received a blood meal, was excised at the pronotum, and both antennae excised between distal segments 12 and 13. Sectioning the final antennal segment allowed for a more reliable connection to the electrodes. The preparation was mounted with an antenna connected to the recording electrode, and the proximal end connected to the reference electrode with the aid of micromanipulators. Glass electrodes were filled with Ringer’s solution [44]. Authentic standards in 10 μL paraffin oil were applied to strips of filter paper at a dose of 1 mg cm^-2^. The filter papers were inserted into a Pasteur pipette, before being allowed to disperse for 1 min to allow the solvent to evaporate. A 5 mL syringe was then attached to the end of the pipette which was depressed to expel the VOC’s into a purified airstream at a flow rate of 1 L min^-1^ through a glass tube (I.D = 12 mm) and over the prepared antennae. The resulting peaks were consistent against a standard of paraffin oil. The EAG equipment consisted of a 10x gain universal probe (Syntech, Netherlands) and an IDAC 2 Signal A and an IDAC 2 Signal Acquisition Processor (Syntech). Data were analysed with EAG-Pro Version 2 software (Syntech, USA). Paraffin oil was tested at the beginning of each experiment and used to normalise the recordings to the largest response in each set. Ten recordings were made to each test compound using three insects.

### 2.6 Single chemical olfactometry

Each of the chemicals to which mosquitoes were responsive, identified from the EAG assays, were assayed separately. Experiments were carried out using the same format as in experiments using essential oil extracts, with each chemical assayed at three differing concentrations; 1, 0.5 and 0.1 mg cm^-2^, in order to determine minimum possible concentrations for repellency to be confirmed. Hexane was used to dilute each of the chemicals tested.

### 2.7 Dual-port olfactometry of repellent blends

The five most repellent chemicals were assayed in combinations of three, four and five components to determine the most repellent blend using the dual-choice olfactometer method outlined above. In these trials the concentrations of each chemical in the blend corresponded to 0.5 mg cm^-2^. Thus, a blend consisting of three, four, or five components had concentrations of 1.5 mg cm^-2^, 2 mg cm^-2^ and 2.5 mg cm^-2^, respectively.

### 2.8 Evaluation of most effective repellent blends using the BG cage test

The two most repellent blends identified by olfactometry were blends 3 and 4, which were investigated further using the Biogents (BG) cage test as outlined in Obermayr *et al*. 2010 [45], based on the guideline published by the American Environmental Protection Agency [46] and in accord with WHO recommendations [40].

Blend 3 consisted of (75 mg) borneol, (75mg) borneol acetate, (75 μL) eugenol, and (75 μL) isoeugenol. Blend 4 was the same as blend 3 but also contained (75 mg) camphor. Both blends were diluted in pure ethanol (1:1) and were dispensed from 30 x 30 mm condensed cellulose pads. Three different volumes (50 μL, 100 μL and 200 μL) of the blends were applied to the pads which were stored in sealed foil bags and stored at 4°C until required. Controls consisted of pads containing 200 μL of ethanol or commercial repellents, DEET, IR3535, PMD, and Picaridin. To determine the robustness of the actives, the blends were assayed against a different population of *Ae. aegypti* and two other mosquito vectors, *Cx. quinquefasciatus* and *An. gambiae*. 30 female mosquitoes that exhibited host-seeking behaviour and had never received a blood-meal were released into BG test cages (41 x 41 x 16 cm). The repellent dispenser was placed on the back of the hand of a mosquito attractive volunteer and their hand placed under the mesh port of the cage. The efficacy of the repellent was measured by counting the number of mosquitoes that landed and probed on the mesh over a 5 minute period [47] and the results compared with the negative controls (i.e. cellulose pad alone or one treated with ethanol). This study was repeated at least 3 times for each mosquito species and repellent.

### 2.9 Statistical analyses

Results from olfactometry assays were treated as positive or negative, based on captured insects in the test port at the end of the experiment. Mean responses were analysed using ANOVA, with all data arcsine transformed to assume ANOVA assumptions of homogeneity of variance. Electrophysiological responses were analysed using EAGPro Version 2 software (Syntech). Data were square root transformed to obtain homogeneity of variance and analysed using ANOVA. Means were compared using Tukeys *post-hoc* test. All statistics were calculated using SPSS v22. (IBM Corportation, USA).

For the BG cage test, the numbers of mosquitoes landing were treated as count data and analysed using a Generalised Linear Model (GLM) with quasi-Poisson error distribution and square root link function. Mosquito species, and substance treatment were included as interacting, categorical explanatory variables. Where appropriate, concentration was included as a further, fully interacting covariate. Statistical significance of explanatory terms was assessed using likelihood ratio tests. All analysis was performed using R version 3.6.1 [48].

## 3. Results

### 3.1 Essential oil olfactometry

**Fig 1:**
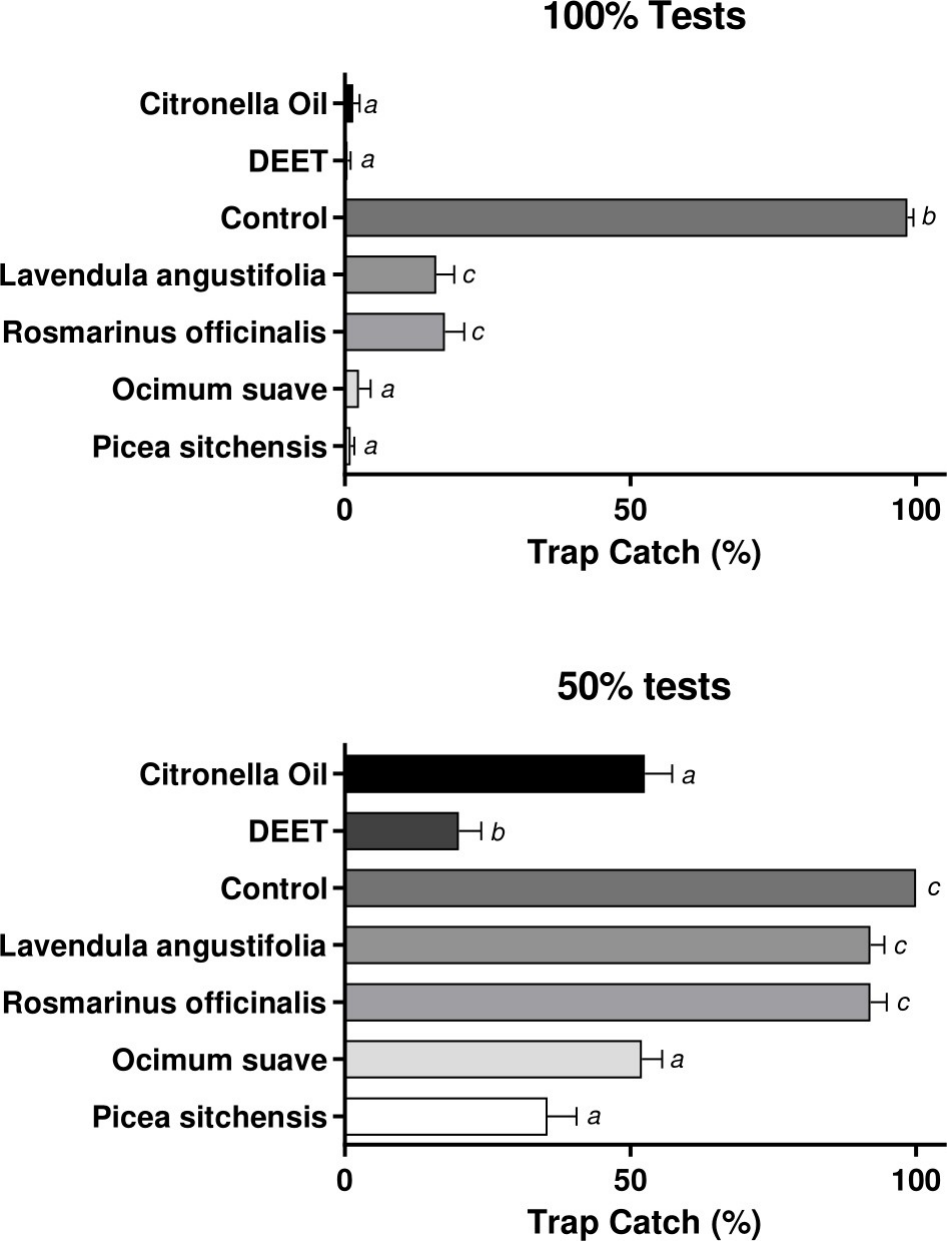
Differential test-arm catches in dual-port olfactometry of plant essential oil extracts at 100% and 50% concentrations. *Aedes aegypti* responses to sock-bait human odours treated with essential oils from 4 plant extracts; *Lavendula angustifolia*, *Rosmarinus officinalis*, *Ocimum suave and Picea sitchensis*. Negative control was an untreated human-odour-baited sock, positive controls were socks treated with the commercially produced repellents DEET and Citronella Oil. Data presented as mean ± 95% CI., treatments labelled with different letters are significantly different (p < 0.05) from each other, those with the same letter denotation represent non-significant results in Tukey’s *post-hoc* multiple comparison analysis.

Female *Ae. aegypti* were highly attracted to socks emanating human foot odours, resulting in 100% trap capture (Fig 1). Essential oils of all test plants were highly repellent at the 100% and 75% concentrations with the differences significant as compared to controls (Fig 1, F_(6,63)_ = 863.325, p <0.001 and F_(6,63)_ = 79.695, p = <0.001 respectively). Of the four test plants, essential oil of *P. sitchensis* was the most repellent, showing no difference to the DEET positive control at both 100% and 50% concentrations (p = 1.000 and p = 0.483, respectively). The second most repellent was *O. suave* which was similar to DEET at 100% (p = 0.998), but less efficacious at 75% (p = 0.004) (Fig 1). Repellency of *P. sitchensis* and *O. suave* essential oils was similar to citronella even at 75% (*p* = 0.249 and *p* = 1.000) (Fig 1). of *L. angustifolia* and *R. officinalis* extracts were similar (p = 0.11 and p = 0.04, respectively) to controls at the 75% concentration. All other assays were significantly different from negative controls (p = <0.001).

### 3.2 Electroantennography

**Fig 2:**
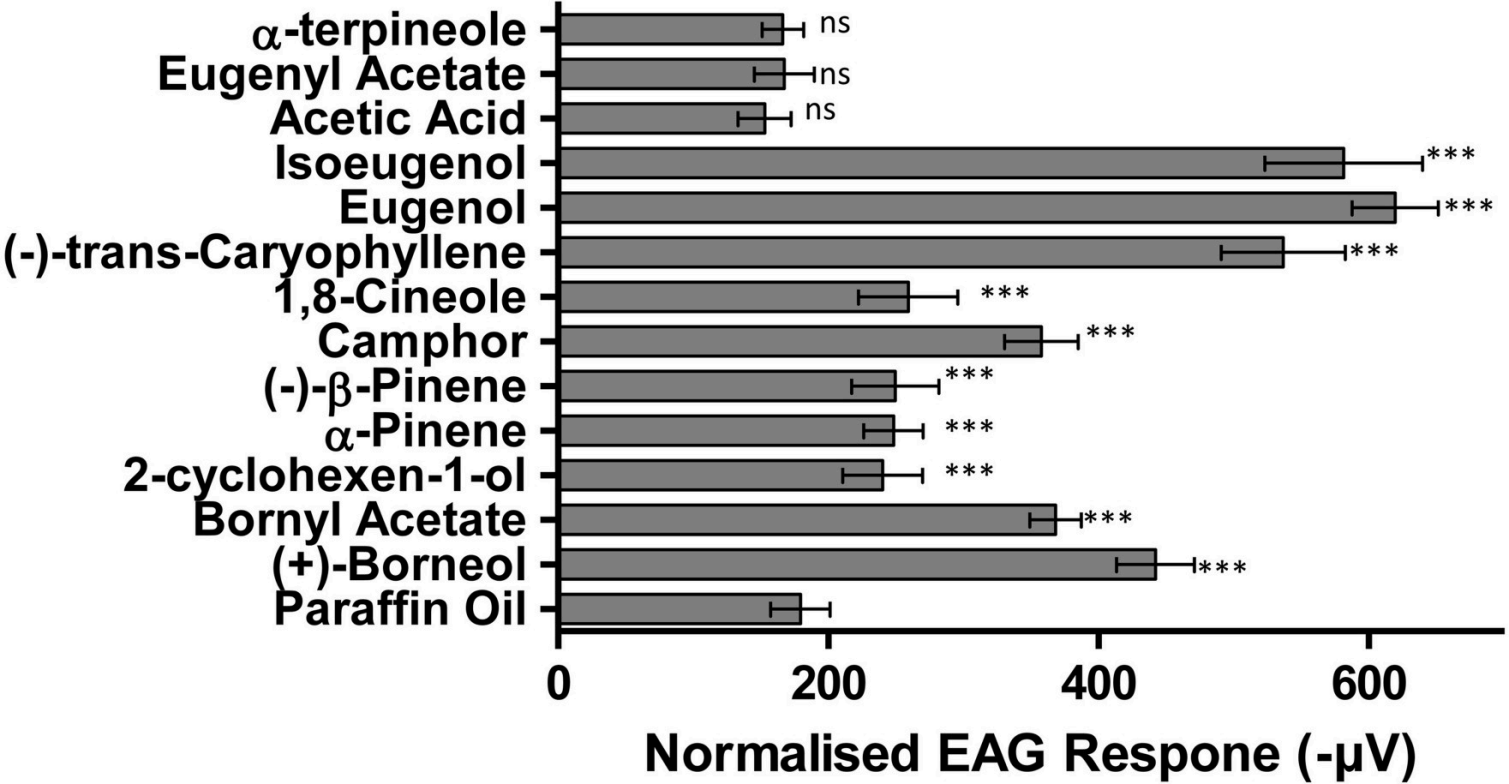
Mean electroantennogram (EAG) responses (normalised to paraffin oil) of *Aedes aegypti* to 13 spruce plant VOC’s. Electrophysiological responses of *Aedes aegypti* to spruce-derived VOC’s. Electrophysiological data collected as female antennal response to 1 mg doses of each treatment VOC formulated in 10 μL paraffin oil on filter paper. Control experimental antennae were exposed to 10 μL paraffin oil only. Data presented as mean ± 95% CI. (*** = *p*<0.001; ** = *p*<0.01; * = *p*<0.05; ns = not significant).

Of the 13 compounds tested for electrophysiological response, 10 ((+)-borneol, (-)-bornyl acetate, camphor, 1,8 cineole, 2-cyclohexen-1-ol, eugenol, isoeugenol, α-pinene, β-pinene, α-terpineol, (-)-transcaryophelene) produced a significant electrophysiological response compared to hexane controls (Fig 2. F_(13,126)_ = 327.435, p = <0.01). The excitatory response elicited by α-terpineole, eugenyl acetate and acetic acid did not differ significantly from the control (Fig 2. p = 1.000, p = 0.999 & p = 0.384, respectively).

### 3.3 Single chemical olfactometry

**Fig 3:**
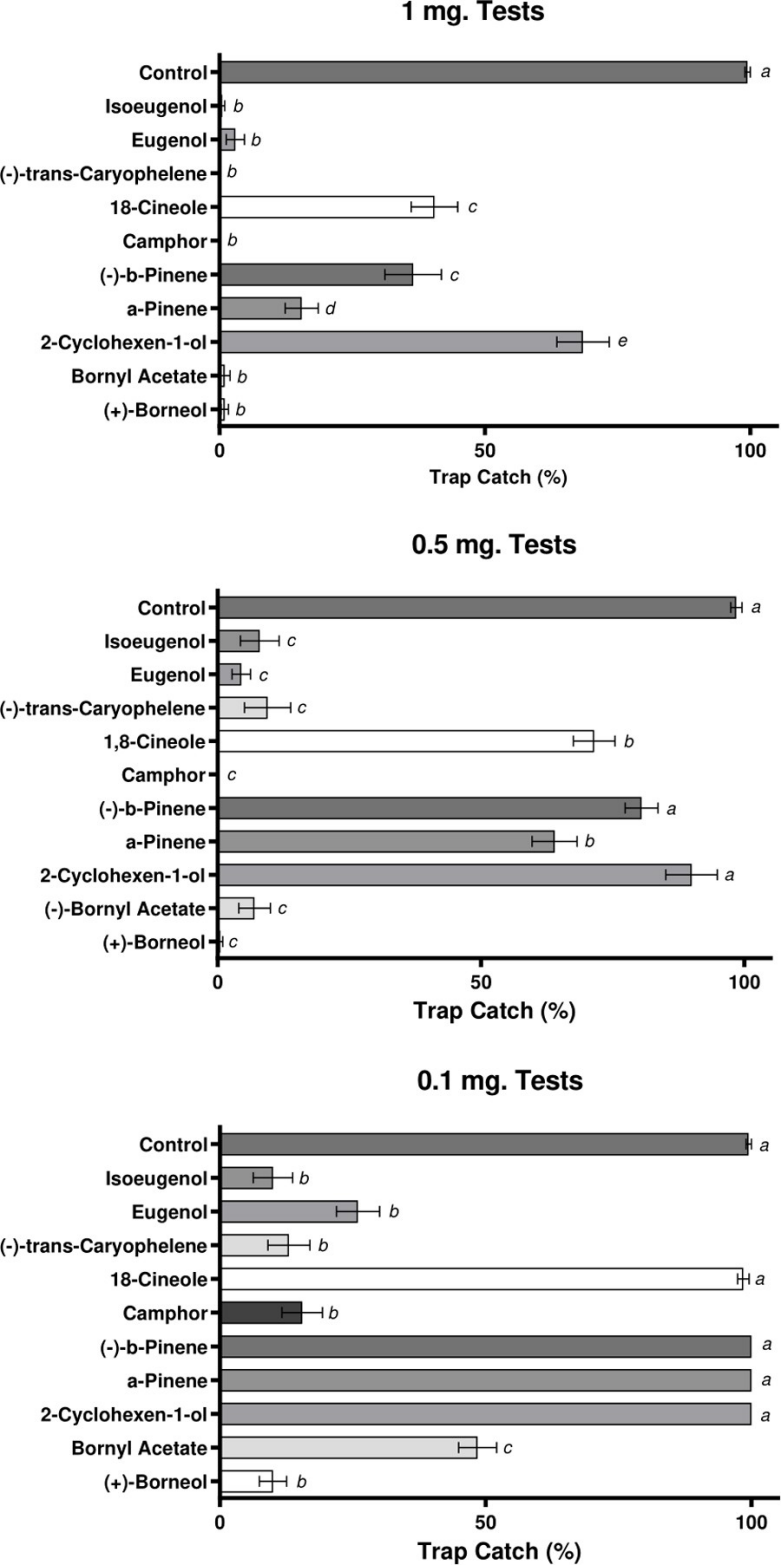
Differential repulsion from human odours under the presence of varied concentrations of each tested spruce volatiles. *Aedes aegypti* responses to 1, 0.5 and 0.1 mg cm^-2^of each of the electrophysiologically active spruce VOC’s applied to human-odour-baited nylon socks in dual-port olfactometric experiments. Data presented as mean ± 95% CI., treatments labelled with different letters are significantly different (p < 0.05) from each other, those with the same letter denotation represent non-significant results in Tukey’s *post-hoc* multiple comparison analysis.

The ten excitatory compounds identified by EAG differed in their repellency and at 1 mg cm^-2^ they were significantly more repellent than the controls (Fig 3. F_(10,99)_ = 63.147, p = <0.001). At 0.5 mg cm^-2^ compounds were also significantly repellent as compared to negative controls (F_(10,99)_ = 44.652, p = <0.001); with the exceptions of 2-cyclohexen-1-ol (p = 1.000) and β-pinene (p = 0.157). At 0.1 mg cm^-2^, only (+)-borneol, (-)-bornyl acetate, camphor, (-)-transcaryophelene, eugenol and isoeugenol were significantly repellent (Fig 3. F_(10,99)_ = 154.125, p = <0.001).

### 3.4 Dual-choice olfactometry for optimised chemical blends

**Fig 4:**
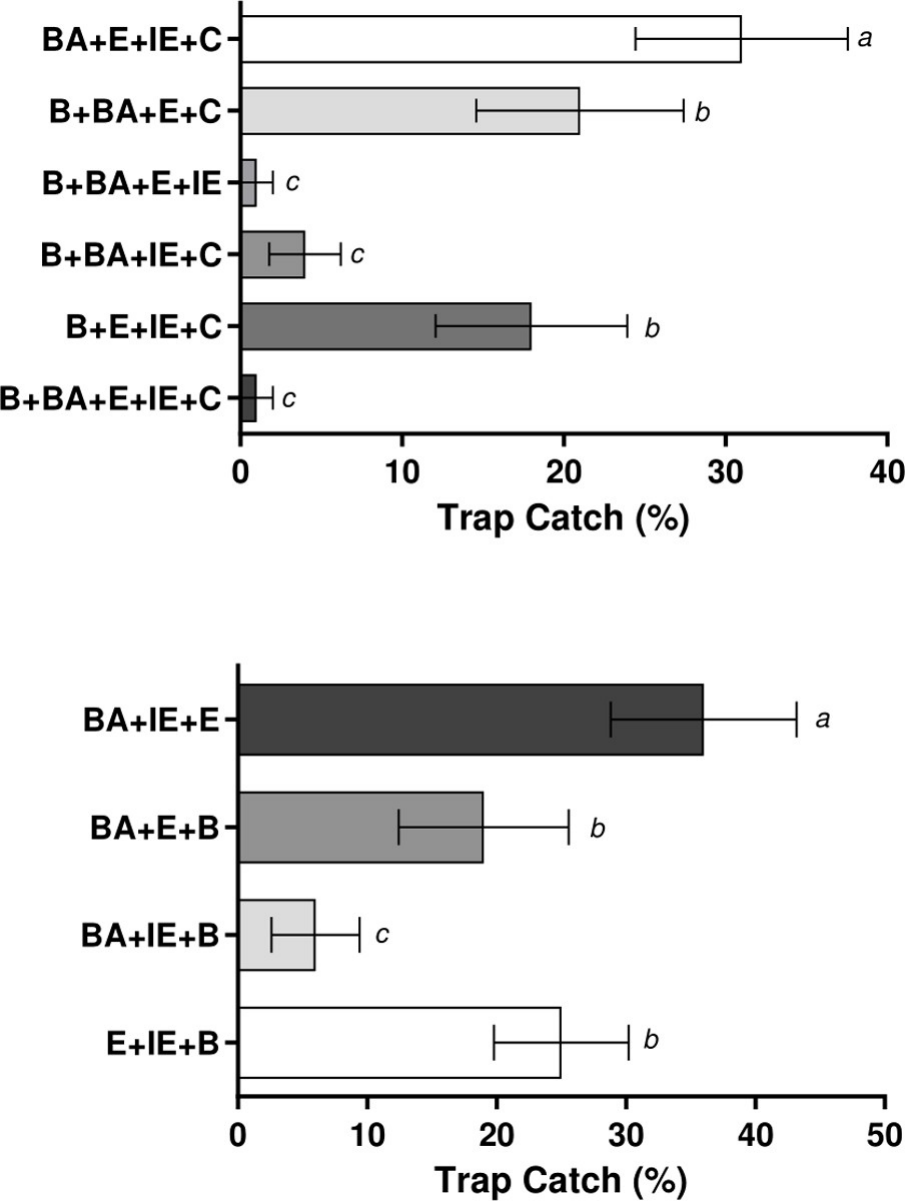
Different levels of trap catches when human odours treated with reductive blends of repellent compounds. Differences in capture rate of adult female *Aedes aegypti* mosquitoes exposed to human-odour-baited socks treated with different blends of the most repellent chemicals found in previous assays. Repulsion as compared to controls was measured. Data presented as mean value ± 95% CI., treatments labelled with different letters are significantly different (p < 0.05) from each other, those with the same letter denotation represent non-significant results in Tukey’s *post-hoc* multiple comparison analysis.

Blends of the most repellent compounds revealed significant differences (F_(5,54)_ = 8.572, p *=* <0.001). A blend of (+)-borneol, (-)-bornyl acetate, eugenol, isoeugenol and camphor was consistently repellent (Fig 4. p = 1.000). Removal of camphor from this blend had no adverse effect on overall repellency rates; (p = 0.989). Some of the other camphor free blends also had significant repellent properties (F_(3,36)_ = 6.912, p = <0.001), notably a blend of (+)-borneol, (-)-bornyl acetate and isoeugenol (Fig 4). Removal of (-)-bornyl acetate significantly increased trap catches, illustrating a drop in repellency whereas omission of eugenol from the mix of all 5 chemicals had little effect. Of the blends assessed the two most repellent were blends 3 ((+)-borneol, (-)-bornyl acetate, eugenol, isoeugenol) and 4 ((+)-borneol, (-)-bornyl acetate, eugenol, isoeugenol and camphor).

### 3.5. BG-test

**Fig 5:**
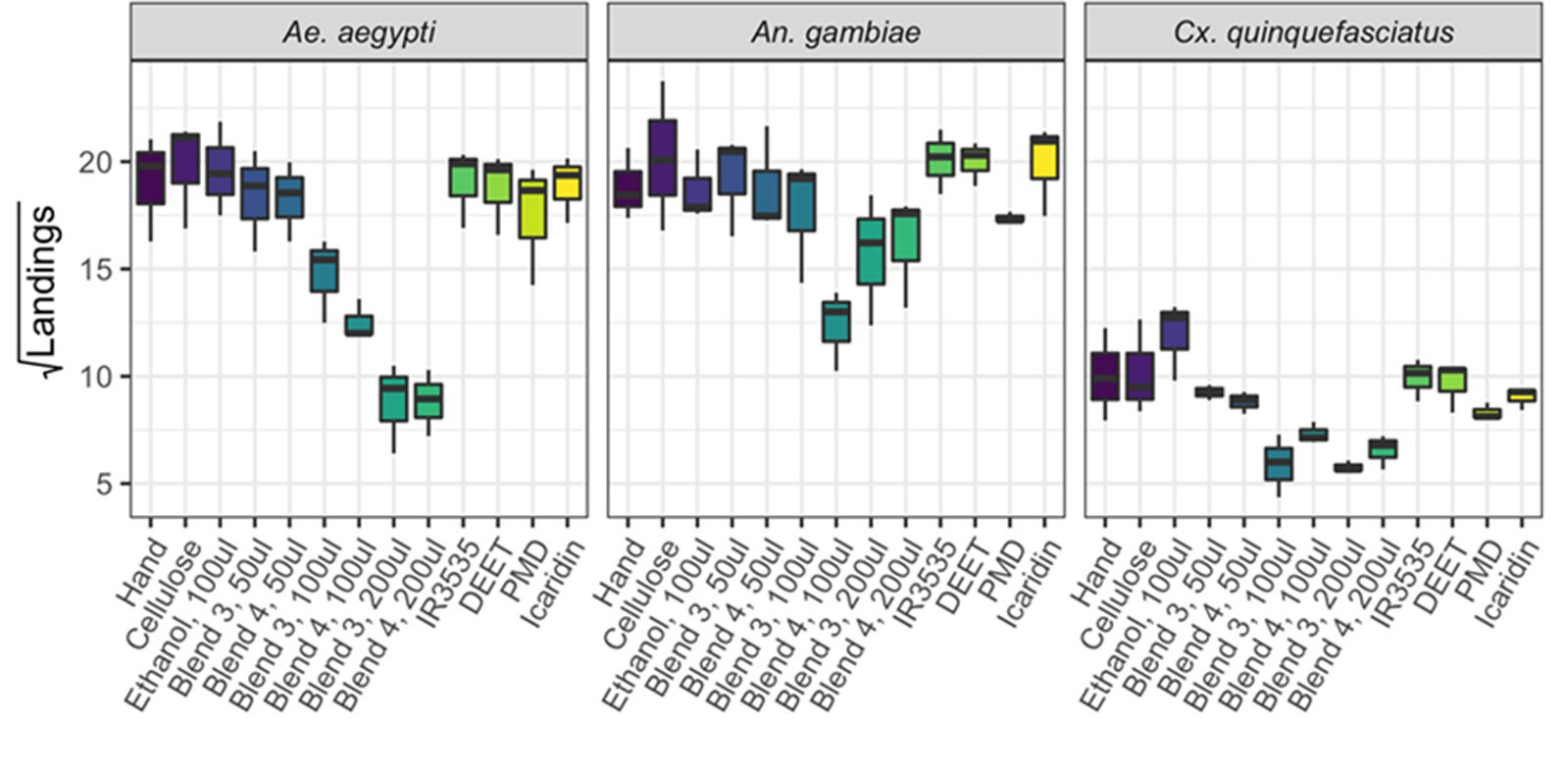
Differences in landing counts elicited by application of blended semiochemicals in BG-cage tests. Boxplots showing the numbers of mosquito landings in five minutes, following treatment with repellent blends 3 and 4 (Note square root scale) in BG-cage tests against adults of three mosquito species: *Aedes aegypti* (left-hand panel), *Anopheles gambiae* (mid panel) and *Culex quinquefasciatus* (right-hand panel). Human arms were presented as bait with a cellulose pad containing treatments placed near the arm. All treatments included a human hand as bait including as a negative control, the second negative control included the human hand and cellulose pad but no treatment. Positive controls included DEET, PMD and Icaridin. Midlines show median values; boxes span inter-quartile range, with Tukey whiskers.

The BG test confirmed that blends 3 and 4 were highly repellent to *Ae. aegypti* (Fig 5). For all mosquito species tested, there was clear avoidance of the mesh immediately above the cellulose pads treated with these blends. Blend 4 appeared slightly more efficacious than blend 3 against *Ae. aegypti* and *An. gambiae*, particularly at the intermediate dose of 100 μl (Fig 5, left-hand and middle panels). The reverse was apparent in *Cx. quinquefasciatus*, with blend 3 reducing landings more than blend 4 (Fig 5, right-hand panel). However, the small sample size in these exploratory trials (n = 3) resulted in the differences between blends not being statistically significant (Likelihood Ratio = 1.15, p = 0.29).

Increasing concentration of blends 3 and 4 reduced the landing rate in all three of the mosquito species. However, the strongest repellency was found for *Ae. aegypti* (Estimate (SE) = -0.06 (0.01), t = -7.69, p < 0.01), while *An. gambiae* (Estimate (SE) = -0.02 (0.01), t = -2.07, p = 0.04) and *Cx. quinquefasciatus* (Estimate (SE) = -0.02 (0.01), t = -2.15, p = 0.04) were less susceptible (Fig 5).

The commercial products (IR3535, DEET, PMD, and Icaridin) produced very low levels of repellence (Fig 5), with none showing statistically significant differences to cellulose pad controls (Table 1). Interestingly, ethanol seemed to be an attractant for *Cx. quinquefasciatus* (Fig 5. √(difference) = 1.83), but this was not statistically significant with n = 3 (t = 1.21, p = 0.23). There was much less evidence that ethanol was an attractant for *Ae. aegypti* (√(difference) = 0.53, t = 0.35, p = 0.73) or *An. gambiae* (√(difference) = -0.14, t = 0.09, p = 0.93). Blends 3 and 4 were significantly better repellents at 200 μl than the commercial products, with the exception of PMD in *An. gambiae* and *Cx. quinquefasciatus* (Tables 2 and 3).

**Table 1:** Repellent effects of cellulose pads treated with commercial products versus untreated cellulose pad controls. Components of table cells are √(mean difference) (t-value, p-value), where a negative difference indicates a reduced number of landings compared to untreated cellulose pad controls.

	*Ae. aegypti*	*An. gambiae*	*Cx. quinquefasciatus*
IR3535	-0.04 (t = 0.03, p = 0.98)	1.24 (t = 0.82, p = 0.42)	-0.23 (t = 0.15, p = 0.88)
DEET	-0.31 (t = 0.20, p = 0.84)	1.16 (t = 0.76, p = 0.45)	-0.47 (t = 0.31, p = 0.76)
PMD	-1.49 (t = 0.98, p = 0.33)	-1.49 (t = 0.98, p = 0.33)	-1.86 (t = 1.22, p = 0.22)
Icaridin	-0.22 (t = 0.14, p = 0.89)	1.14 (t = 0.75, p = 0.45)	-1.14 (t = 0.76, p = 0.45)

**Table 2:** Repellent effect of cellulose pads treated with commercial products versus cellulose pads treated with blend 3. Components of table cells are √(mean difference) (t-value, p-value), where a negative difference indicates a reduced number of landings compared to untreated cellulose pad controls. Shown in bold where blend 3 is significantly more repellent than commercial products at α = 0.05.

	*Ae. aegypti*	*An. gambiae*	*Cx. quinquefasciatus*
IR3535	**10.2 (t = 6.70, p <0.01)**	**4.23 (t = 2.79, p <0.01)**	**4.18, t = 2.76, p = 0.01**
DEET	**9.90 (t = 6.53, p <0.01)**	**4.15 (t = 2.74, p <0.01)**	**3.94 (t = 2.15, p = 0.03)**
PMD	**8.72 (t = 5.75, p <0.01)**	1.50 (t = 0.99, p = 0.32)	2.55 (t = 1.68, p = 0.10)
Icaridin	**9.99 (t = 6.59, p <0.01)**	**4.13 (t = 2.73, p <0.01)**	**3.26 (t = 2.15, p = 0.03)**

**Table 3:** Repellent effect of cellulose pads treated with commercial products versus cellulose pads treated with blend 4. Components of table cells are √(mean difference) (t-value, p-value), where a negative difference indicates a reduced number of landings compared to untreated cellulose pad controls. Shown in bold where blend 4 is significantly more repellent than commercial products at α = 0.05.

	*Ae. aegypti*	*An. gambiae*	*Cx. quinquefasciatus*
IR3535	**10.2 (t = 6.73, p <0.01)**	**3.74 (t = 2.47, p = 0.02)**	**3.37 (t = 2.22, p = 0.03)**
DEET	**9.93 (t = 6.55, p <0.01)**	**3.65 (t = 2.41, p = 0.02)**	**3.13 (t = 2.06, p = 0.04)**
PMD	**8.75 (t = 5.78, p <0.01)**	1.01 (t = 0.67, p = 0.51)	1.74 (t = 1.15, p = 0.25)
Icaridin	**10.0 (t = 6.61, p <0.01)**	**3.64 (t = 2.40, p = 0.02)**	**2.45 (t = 1.62, p = 0.11)**

## 4. Discussion

This study has identified novel blends of compounds from Sitka spruce that were highly repellent to host seeking *Ae. aegypti*, *Cx. quinquefasicatus and An. gambiae*. Spruce extracts were similar or superior to the extracts of lavender, murihani and rosemary, adding to the increasing list of plants whose extracts have been shown to repel mosquitoes [49–51]. EAG revealed that mosquitoes respond to a wide range of plant derived excitatory compounds, corroborating the findings of others [27]. Olfactory detection of compounds does not, however, infer specific behavioural response, therefore olfactometric methods were used to establish (+)-borneol, (-)-bornyl acetate, eugenol, isoeugenol and camphor as repellent compounds. The disparity between electrophysiological and behavioural responses is well documented [38, 52]. The novo blends (3 and 4) were significantly more effective than leading commercial repellents in olfactometer and arm-in-cage tests.

Olfactometric assays determined that the essential oils of sitka spruce, rosemary, murihani and lavender were all repellent to mosquitoes, corroborating the findings of others [34, 36, 53]. While some studies have found conifer oils to be effective larvicides [29], this is the first study to demonstrate the repellent activity of spruce oil against adult *Aedes* mosquitoes. The broad utility of plant essential oils in mosquito control is clear through the range of larvicidal and semiochemical actions demonstrated against both adult and larval mosquitoes [1, 54–56], however, while many studies have progressed to identify useful attractant semiochemicals from plants [56, 57], relatively few have attempted to elucidate repellent compounds [1, 58].

(+)-Borneol, (-)-bornyl acetate, eugenol, isoeugenol and camphor are present in extracts of a broad range of plant species in various ratios and quantities [59–61]. Individually, they are known to influence the behaviour of a wide range of insect species, some being repellent and others being attractants depending on the dose and insect species in question [62–66]. It is of note that those attracted to the compounds outwardly tend to be herbivorous, and those repelled are blood feeding; given the relative conservation of olfactory stimuli in blood feeding arthropods, it is possible that the blends described herein will have functions beyond mosquito control. This aspect may also explain the similar functionality of the overall blend, despite clear differences in response between species; interactions between components of the blend, while not synergistic, interact in a manner so as to increase the breadth of potential target species. Some of the blend components have previously been shown to repel mosquitoes, but weakly compared to other plant components. For example, Hwang et al. [67] identified numerous repellents from mugwort (*Artemisia vulgaris* [Asterales: Asteraceae]), including borneol and camphor, but established terpinen-4-ol to be the most repellent against *Aedes aegypti*. This pattern is seen for many other studies where researchers focussed on alternative compounds [68, 69]. Most researchers have focussed on single compounds, presumably because they are easier to register with regulatory authorities. Also no advantage is seen in preparing mixtures of repellents unless they act synergistically and can be used in significantly lower concentrations but equally important is the smell and safety of the actives. In the current study, no obvious synergy was observed for blends 3 and 4 but the bouquet was pleasant to smell, indeed, they are exploited in the perfume industry [70].

Repellency of both blends 3 and 4 was dose dependent but the response of the three mosquito genera was not identical suggesting differences in olfaction. Lui et al. [71] found that the sensilla of *Cx. quinquefasciatus* showed significant excitatory responses to certain types of repellents, especially terpene-derived chemicals such as eucalyptol, α-pinene, and camphor, in a dose-dependent manner. Females of all three vector genera, *Aedes*, *Culex* and *Anopheles* respond to human odours (carbon dioxide, fatty acids, ammonia), at both long and close range, during host-seeking for a blood meal but also to a whole range of other chemical cues linked to their ecology such as plant and microbial volatiles which help locate suitable oviposition sites [55, 72]. Equally important are floral volatiles (e.g. terpenes, aromatic aldehydes) which enable male and female mosquitoes to locate nectar rich flowers as nectar-feeding enhances their survival and reproductive success [32, 73]. Interestingly, *Ae. aegypti* and *Cx. quinquefasciatus* were more responsive to blends 3 and 4 than *An. gambiae*. Presumably, these differences are linked to *Cx. quinquefaciatus* and *Ae. aegypti* genomes have a significantly larger number of odour binding protein (OBP) genes than *An. gambiae* [74]. Furthermore, *Ae. aegypti* and *Cx. quinquefasciatus* belong to the same subfamily Culicinae, whereas *An. gambiae* belongs to the Anophelinae, with the two subfamilies having diverged ca. 120 million years ago [75].

While weak spatial repellency has been demonstrated under laboratory conditions, further work will be required to assess the efficacy of the compounds under ‘field’ and ‘semi field’ settings, whereby the real-term efficiency of the products can be evaluated. Furthermore, given the high conservancy of olfactory receptors across arthropod species, the scope of usage for the compounds may be much greater than mosquitoes alone.

## 5. Conclusions

Two blends of sitka spruce derived volatile compounds were formulated to form an efficacious repellent blend that has activity against three important mosquito vector species; *An. gambiae*, *Ae. aegypti* and *Cx. quinquefasciatus*. The blends were more effective than currently available commercial repellents and provided weak spatial repellency under laboratory conditions–an area for which there is a stark lack of products available. Much work will be required to fully assess the real-term efficacy of the products, however, the strength of the blend presented herein demonstrates the value of plant-derived VOCs in the development of novo-repellent products.
